# Sex, Nutrition, and NAFLD: Relevance of Environmental Pollution

**DOI:** 10.3390/nu15102335

**Published:** 2023-05-16

**Authors:** Arianna Dolce, Sara Della Torre

**Affiliations:** Department of Pharmaceutical Sciences, University of Milan, 20133 Milan, Italy

**Keywords:** nutrition, contaminated food, environmental pollution, endocrine disrupting chemicals, climate change, liver, NAFLD, sex and gender differences, fertility, pregnancy

## Abstract

Non-alcoholic fatty liver disease (NAFLD) is the most common form of chronic liver disease and represents an increasing public health issue given the limited treatment options and its association with several other metabolic and inflammatory disorders. The epidemic, still growing prevalence of NAFLD worldwide cannot be merely explained by changes in diet and lifestyle that occurred in the last few decades, nor from their association with genetic and epigenetic risk factors. It is conceivable that environmental pollutants, which act as endocrine and metabolic disruptors, may contribute to the spreading of this pathology due to their ability to enter the food chain and be ingested through contaminated food and water. Given the strict interplay between nutrients and the regulation of hepatic metabolism and reproductive functions in females, pollutant-induced metabolic dysfunctions may be of particular relevance for the female liver, dampening sex differences in NAFLD prevalence. Dietary intake of environmental pollutants can be particularly detrimental during gestation, when endocrine-disrupting chemicals may interfere with the programming of liver metabolism, accounting for the developmental origin of NAFLD in offspring. This review summarizes cause–effect evidence between environmental pollutants and increased incidence of NAFLD and emphasizes the need for further studies in this field.

## 1. Introduction

Non-alcoholic fatty liver disease (NAFLD) is the most common form of chronic liver disease with a 30% prevalence in the general population [1] and represents an increasing public health issue being an independent risk factor for several comorbidities, such as type 2 diabetes (T2D), hypertension, dyslipidemia, atherosclerosis, and cardiovascular diseases (CVDs) among others [2,3,4]. In NAFLD patients, enhanced lipid uptake and de novo lipogenesis (DNL), insufficient fatty acid oxidation (FAO), and reduced lipid secretion lead to excessive accumulation of triglycerides (TG) within hepatocytes [5]. The increased lipid content together with the impaired FAO promotes the generation of reactive oxidative species (ROS) and lipotoxic lipid intermediates within the hepatocytes, fostering oxidative stress and endoplasmic reticulum (ER) stress [6,7,8]. Chronic oxidative stress, in turn, triggers a pro-inflammatory response, mainly mediated by JNK (c-Jun N-terminal kinase) and NF-κB (nuclear factor-κB) signaling pathways, that results in the increased production of pro-inflammatory cytokines (i.e., IL-6, interleukin 6; TNFα, tumor necrosis factor α) by hepatocytes and non-parenchymal cells [7,9,10,11]. The sustained activation of the pro-inflammatory response bolsters a chronic inflammatory state that triggers the recruitment of other immune cells and activates apoptosis and other cell death mechanisms, boosting the progression of NAFLD toward non-alcoholic steatohepatitis (NASH), fibrosis, and hepatocellular carcinoma (HCC) [9,10,11].

Despite the growing public health impact of NAFLD, treatment options remain limited, likely a consequence of the poor understanding of the biological drivers responsible for NAFLD pathogenesis and progression [5,12,13].

## 2. Nutrition, Sex Differences, and NAFLD

In addition to genetic and epigenetic factors [14,15], obesity, dietary habits, lifestyle, and gut microbiota dysbiosis have a major role in NAFLD incidence, development, and progression [5,16,17,18,19,20]. In particular, overnutrition and poor dietary habits trigger insulin resistance (IR) and increase adiposity, favoring lipid deposition in the liver [5,21]. Beyond positive energy intake, consumption of specific nutrients such as saturated and *trans* fatty acids or fructose can be particularly detrimental to liver health, facilitating NAFLD [16,17,22]. Overnutrition and unbalanced diets may induce gut dysbiosis and increase gut permeability, alter the gut–liver axis, and expose the liver to microbiota-derived harmful substances, such as lipopolysaccharide (LPS), ROS, pro-inflammatory cytokines, thereby increasing susceptibility to NAFLD [20].

Compared to men, women show a lower susceptibility to NAFLD, at least until menopause, after which NAFLD prevalence becomes similar for the two sexes [23,24], pointing to the protective effect of estrogens. Accordingly, the incidence of NAFLD is greater in women with reproductive dysfunctions characterized by altered estrogen levels (i.e., polycystic ovary syndrome, PCOS) with respect to fertile women [23,24]. Notably, circulating levels of 17β-estradiol are lower in pre-menopausal, post-menopausal, and PCOS women with NAFLD compared to their control counterparts, while estrogen-based hormone replacement therapy (HRT) reduces the risk of developing NAFLD in post-menopausal women [23,24]. In the liver of fertile female mice, estrogens act mainly through ERα (estrogen receptor alpha), which activity changes according to circulating estrogens, thus modulating the hepatic metabolism to the energy requirements characterizing each reproductive stage [25,26]. In the absence of such an oscillatory activation (e.g., after ovariectomy, OVX), liver metabolic homeostasis is altered, leading to hepatic lipid deposition and inflammation [25,27], a condition resembling the increased incidence of NAFLD observed in post-menopausal women [23,24].

By virtue of its role in the female liver, hepatic estrogen signaling strongly contributes to sex differences in the regulation of hepatic metabolism [28] and in the sex-specific susceptibility to NAFLD [23,24,29]. Hepatic ERα signaling confers to females but not to males the ability to adapt a metabolic response to the excess of dietary lipids, thus limiting liver lipid deposition in a diet-induced mouse model of NAFLD [29]. Notably, such a female-specific and ERα-dependent feature is associated with the ability to preserve the hepatic homeostasis of amino acids (AA), especially of branched-chain amino acids (BCAA) [29], in agreement with studies reporting an impaired BCAA metabolism [30,31] and sex-dimorphic changes in BCAA levels in patients with NAFLD/NASH [32]. Accordingly, BCAA shows the potential to alleviate hepatic steatosis and liver injury in a NASH mouse model [33]. Notably, a dietary formula enriched in essential AA and, especially, in BCAA has been shown to rescue the hepatic transcriptomic profile and limit liver lipid deposition in a mouse model of menopause, an effect that strongly relies on hepatic ERα [27].

This ERα-dependent, female-specific ability to modulate hepatic metabolism according to dietary AA may depend on the strict interplay between metabolism and reproduction gained during evolution by the female liver [18]. In addition to estrogens, indeed, dietary AA activates ERα in the liver of females to promote the progression of the reproductive cycle [17]. By virtue of this mechanism and of the sex-dimorphic expression of hepatic ERα, the regulation of hepatic AA metabolism is significantly different in the liver of the two sexes, and it is strongly affected by the nutritional status [28,29]. Under short-term fasting, liver ERα promotes in females, but not in males, the catabolism of the hepatic AA to preserve lipid synthesis, ensuring the progression of the reproductive cycle [28].

In this view, hepatic ERα may act as a sensor of hormonal and nutritional status and differently accounts for the regulation of hepatic metabolism in the two sexes, thus contributing to sex differences in NAFLD susceptibility [34,35]. Furthermore, ERα might have a role in counteracting NAFLD progression to NASH, as suggested by studies reporting a low expression of ERα in the liver of NASH compared to NAFLD patients [36].

## 3. Nutrition, Environmental Pollutants, and NAFLD

**Figure 1:**
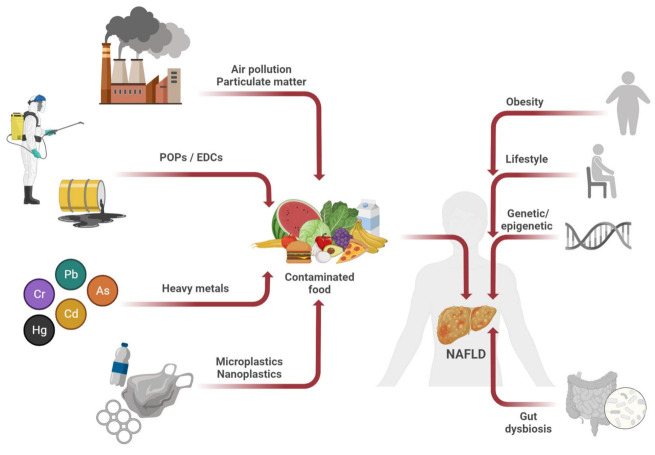
In addition to obesity, lifestyle, genetic and epigenetic factors, and gut microbiota dysbiosis, dietary intake of several environmental pollutants, including persistent organic pollutants (POPs), endocrine disrupting chemicals (EDCs), heavy metals, micro- and nanoplastics promotes NAFLD development and progression. In addition to ingestion, exposure to air particulate matter through inhalation can be another risk factor for developing NAFLD, especially in the context of high urbanization due to the combination with other risk factors, including obesogenic food environment. Figure created with BioRender (https://biorender.com/, accessed on 11 April 2023).

Increasing evidence indicates that dietary intake of several environmental pollutants, including persistent organic pollutants (POPs), endocrine disrupting chemicals (EDCs), heavy metals, and micro- and nanoplastics promotes NAFLD development and progression (Figure 1). In addition to ingestion, exposure to pollutants through inhalation (i.e., particulate matter) can be another risk factor for developing NAFLD, especially in the context of high urbanization due to the combination with other risk factors, including obesogenic food environment, circadian disruption by light at night, and reduced physical activity [16,18,37,38].

### 3.1. Persistent Endocrine Disrupting Chemicals

POPs are long-lived carbon-based organic chemicals that have become a concern for human health, given their large use in agricultural and industrial sectors and their potential to enter the food chain [39]. To this class belong pesticides, including organochlorine pesticides (OCPs) such as DDT (dichlorodiphenyltrichloroethane) and its metabolites; industrial chemicals, including long-chain perfluorinated chemicals such as PFOA (perfluorooctanoic acid) and PFOS (perfluorooctanesulfonic acid), PCBs (polychlorinated biphenyls), and PBDEs (polybrominated diphenyl ethers); by-products of industrial processes, including PCDDs (polychlorinated dibenzo-*p*-dioxins), PCDFs (polychlorinated dibenzofurans), and PAHs (polyaromatic hydrocarbons) [39]. Given their widespread distribution in the environment, their employment in food production (i.e., pesticides), and in food contact materials (i.e., phthalates) from which they can be released, POPs can enter the food chain. It has been estimated that over 90% of human exposure to POPs released into the environment occurs through the consumption of contaminated food [39]. Given their lipophilic nature, POPs can be accumulated especially in the adipose tissue [40,41,42,43], and can be sequestrated in the liver [44], thus leading to metabolic dysfunctions.

**Figure 2:**
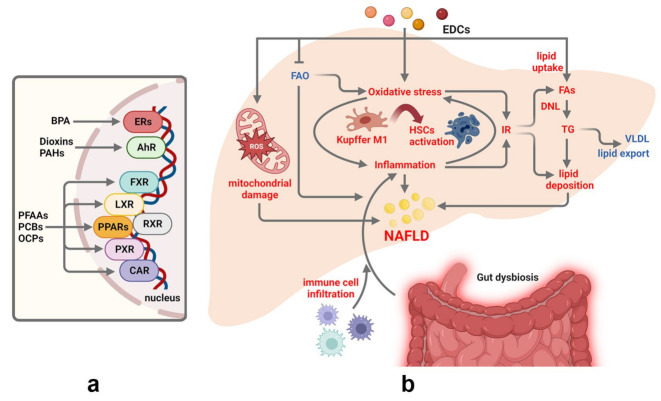
Major effects of persistent EDCs on the main pathways involved in NAFLD development. (**a**) The steatosis-inducing effect of EDCs is mediated by the binding with different nuclear receptors. (**b**) EDC-induced disruption of nuclear receptor signaling promotes lipid accumulation within hepatocytes through the increase in lipid uptake and DNL, the inhibition of FAO, and the reduction of lipid secretion in the form of VLDL. EDCs induce oxidative stress and hepatic inflammation by inducing the polarization of Kupffer cells towards a pro-inflammatory phenotype, increasing immune cell infiltration, and by transforming HSCs into myofibroblast-like cells, thus favoring liver damage and fibrosis. EDCs-induced gut dysbiosis contributes to NAFLD progression by sustaining a pro-inflammatory status. The pathways/factors that increased or decreased are shown in red or blue, respectively. Abbreviations: AhR: hydrocarbon receptor; BPA: Bisphenol A; CAR: constitutive activated receptor; DNL: de novo lipogenesis; ERs: estrogen receptors; FAs: fatty acids; FAO: fatty acid oxidation; FXR: farnesoid X receptor; HSCs: hepatic stellate cells; IR: insulin resistance; Kupffer M1: Kupffer cells with an M1 phenotype; LXR: liver X receptor; OCPs: organochlorine pesticides; PAHs:, polyaromatic hydrocarbons; PCBs: polychlorinated biphenyls; PFAAs: perfluoroalkyl acids; PPARs: peroxisome proliferator-activated receptors; PXR: pregnane X receptor; RXR: retinoid X receptor; TG: triglycerides; VLDL: very-low density lipoproteins. Figure created with BioRender (https://biorender.com/, accessed on 11 May 2023).

Several POPs, including industrial chemicals such as PCBs, plasticizers including phthalates and bisphenol A (BPA), and agricultural pesticides act as EDCs by interfering with hormone signaling and endocrine functions [45,46]. EDCs can disrupt endocrine function in a variety of ways, by interfering with the synthesis, transport, metabolism, and/or receptor binding of hormones, resulting in reproductive, developmental, and metabolic dysfunctions [47,48]. Accumulating evidence suggests that exposure to EDCs may increase NAFLD risk [45,46,49]. The steatosis-inducing effect of EDCs is mostly mediated by the binding with different nuclear receptors (NRs), especially ERα and PPARs (peroxisome proliferator-activated receptors) (Figure 2a). EDC-induced disruption of NR signaling promotes lipid accumulation within hepatocytes through several metabolic alterations, including the increase in lipid uptake, enhanced DNL, inhibition of FAO, and reduction of lipid secretion in the form of VLDL (very-low-density lipoproteins) particles and bile acids [46] (Figure 2b). Several EDCs can contribute to the progression of NAFLD and hepatic inflammation by inducing the production of cytokines and the polarization of Kupffer cells towards a pro-inflammatory phenotype, by increasing hepatocyte proliferation and immune cell infiltration, by promoting the transformation of hepatic stellate cells (HSCs) into myofibroblast-like cells thus favoring apoptosis, liver damage, and fibrosis development [46,50] (Figure 2b).

BPA is an anti-androgen and estrogen-like synthetic chemical, mostly employed in epoxy resins, polymer materials, and plastics; it can be released from food containers and water bottles [51]. Although its use has been banned by several countries, the study of the effects of exposure to BPA is still relevant and representative of other chemical compounds with potential estrogenic activity. In addition to cancer, hormonal disruption, immunosuppression, and infertility, BPA is linked to obesity and several metabolic disorders, including NAFLD [46,51,52,53]. A positive association has been found between high urinary levels of BPA and increased incidence of NAFLD [54,55]. In vitro and animal-based studies have demonstrated that BPA stimulates TG accumulation in liver cells by upregulating the expression of the genes involved in DNL and IR, and by disrupting lipid homeostasis [46,56,57,58,59]. Through the generation of oxidative stress [60] and of a pro-inflammatory status that involves the polarization of Kupffer cells toward an M1 phenotype [50], and through the cross-talk among these two signaling pathways [61], BPA further promotes the progression of NAFLD and liver injury [46,59]. When coupled with a high-fat diet (HFD), BPA exposure worsens hepatic steatosis by stimulating ROS-induced overexpression of *Cd36* (fatty acid translocase) in the mouse liver, thus facilitating lipid uptake [62]. In rodents, BPA may lead to hepatic steatosis and hepatotoxicity also by inducing gut microbiota dysbiosis and activating the gut-liver axis [63,64]. In OVX mice fed with HFD, BPA exposure aggravates hepatic steatosis by upregulating genes involved in DNL, fatty acid β-oxidation, and mitochondrial uncoupling, lowering lipid export with decreased expression of *Mttp* (microsomal triglyceride transport protein), and inducing ER stress, resulting in hepatic lipotoxicity, collagen deposition, and fibrosis [65].

Perfluoroalkyl acids (PFAAs, i.e., PFOA; perfluorononanoic acid, PFNA) are persistent, active chemicals in the environment, that can be ingested also through contaminated food and water [66], and, due to their exceptional stability to degradation, are slowly eliminated by the human body [67]. Environmental exposure to high levels of PFAAs increases the risk of developing hepatic steatosis and NAFLD in adults and children [68,69] and promotes liver injury [70]. PFAAs administration induces hepatic steatosis in rodents [71,72,73] and increases lipogenic gene expression signatures in cultured hepatocytes [74]. In male mice, PFOS induces fatty liver in a dose and time-dependent manner, by upregulating *Cd36* and *Lpl* (lipoprotein lipase), inhibiting mitochondrial β-oxidation, and causing a shift of the hepatic proteome [71,72]. In HFD-fed mice, PFOS facilitates liver inflammation and steatosis through the activation of NLRP3 (NLR family pyrin domain containing 3) inflammasome that mediates hepatocyte pyroptosis [73]. PFOA exacerbates HFD-induced hepatotoxicity and lipid accumulation in the liver through the activation of peroxisome proliferator-activated receptor α (PPARα) [75]. Other studies have demonstrated that the co-administration of PFOS and PFNA with HFD reduces hepatic lipid deposition, a paradoxical effect likely due to the reduced expression of hepatic PFAAs uptake transporters and organic anion transporter proteins in the presence of HFD [76]. In rodents, PFAAs-induced liver steatosis and toxicity have been mainly attributed to PPARα, which activation leads to increased expression of genes involved in FAO, lipogenesis (*Srebf1*, sterol regulatory element-binding transcription factor 1; *Fasn,* fatty acid synthase), and lipid uptake (*Cd36*) [77]. However, studies performed in PPARα KO mice have demonstrated that other nuclear receptors and transcription factors, including ERα, peroxisome proliferator-activated receptor γ (PPARγ), constitutive activated receptor (CAR), liver X receptor (LXRα), pregnane X receptor (PXR), farnesoid X receptor (FXR), and hepatocyte nuclear factor 4α (HNF4α), may account for PFAAs effects in the liver (Figure 2a), resulting in a metabolic shift from carbohydrate metabolism to fatty acid oxidation and in hepatic TG accumulation [78,79]. Likely a consequence of sex differences in their pharmacokinetics and tissue distribution [80,81], PFAAs differently account for steatosis and toxicity in the liver of the two sexes [82,83,84], with females showing a positive association with NAFLD and increased hepatic inflammation and injury compared to males [83,84].

Several studies have reported a correlation between exposure to pesticides such as DDT and its metabolites and metabolic disorders, including NAFLD [85,86,87,88], likely because the liver is the main target for these toxicants. Retrospective analysis of the U.S. National Health and Nutrition Examination Survey 2003–2004 has indicated that exposure to several OCPs, especially to oxychlordane, is strongly associated with hepatic steatosis and altered levels of hepatic enzymes [89]. Although the underlying mechanisms have not yet been fully clarified, multi-omic analysis has suggested that OCPs favor NAFLD by altering the hepatic expression of genes involved in liver steatosis, PPAR signaling and fatty acid metabolism, inflammation, and necrosis [90]. Notably, some altered signaling pathways are common among different OCPs or a mixture of OCPs, while others are typical for a specific chemical [90]. For example, DDT leads to an over-expression of genes and transcription factors known to be linked to IR, cell death, and necrosis; in addition, DDT increases the hepatic levels of arginine, proline, and putrescine, and lowers the levels of urea and ornithine, impacting on liver regeneration process and boosting NAFLD progression [90]. In addition to these direct effects on the liver, OCPs can trigger NAFLD also by causing gut microbiota dysbiosis [91] and inhibiting thermogenesis in brown adipose tissue (BAT) [92]. In treated mice, insecticides such as fipronil (a phenylpyrazole commonly used in agricultural and veterinary fields) and thiamethoxam (a major compound of neonicotinoids) alter lipid metabolism by increasing *Pparγ* and *Fasn* expression and promote the generation of oxidative stress and inflammation by decreasing *Pparα* and *Gnmt* (glycine n-methyltransferase), ultimately contributing to NAFLD and liver injury [93,94]. Fungicides such as myclobutanil and mancozeb contribute to hepatic lipid deposition, cellular damage, and NAFLD development and progression [95,96,97]. In mouse liver, the effects of Myclobutanil can be differently mediated by the signaling of FXR depending on nutritional conditions [95]. In HepG2 cells, myclobutanil, mancozeb, and other fungicides such as tributyltin (TBT) induce steatosis also via retinoid X receptor alpha (RXRα), and hepatotoxicity by decreasing the expression of anti-apoptotic markers [96,97,98].

Dioxins consist of a group of organochlorines that include PCDDs, PCDFs, PCBs, and other related compounds that persist in the environment and accumulate in the food chain [99,100]. Dioxins can be produced from natural sources (i.e., volcanoes, forest fires), but are mostly derived from human industrial activities, especially as by-products of organochloride and chlorine-containing substances such as polyvinyl chloride (PVC). Dioxins are neither readily metabolized nor excreted, with a mean half-life of several years in humans, and bioaccumulate in fat tissues due to their lipophilic nature [101]. Several epidemiological studies have reported an increased risk of developing cancers, nervous system degeneration, immune damage, thyroid disease, metabolic disorders, and reproductive and sexual development disorders in the general population as well as in subgroups such as Vietnam War veterans heavily exposed to dioxins [100,102]. With respect to liver health, TCDD (2, 3, 7, 8-tetrachlorodibenzo-*p*-dioxin), the most toxic dioxin, has been shown to alter blood and hepatic lipid levels, disrupt bile synthesis, impair the microbiome, and strongly contribute to NAFLD development and progression through the activation of aryl hydrocarbon receptor (AhR) [103,104,105,106,107]. Several other dioxins, including PCBs, have become a major concern for liver health, given their strong association with IR and NAFLD/NASH, especially under diet-inducing obesogenic conditions [86,108,109,110,111]. Mice show significant differences in hepatic transcriptomic response to TCDD, with greater changes in the liver of males than females, likely due to sex differences in hepatic detoxification and to a complex interaction between the AhR and sex hormone receptors [112]. Notably, TCDD-induced activation of AhR leads to the disruption of hepatic sexual dimorphism in mice [113] and to a sex-specific induction of genes such as *Car* and proteins such as FMO3 (flavin-containing dimethylaniline monooxygenase 3) only in the livers of male mice [112,114]. Conversely, prolonged exposure to low doses of dioxin impairs metabolic adaptability to HFD only in female mice, which show hyperglycemia and impaired glucose-induced plasma insulin [115]. Furthermore, hepatic metabolic alterations could be indirect and secondary to sex-dependent glucocorticoid signaling disturbances and clock-related gene expression modifications caused by dioxins in adipose tissue [116].

PAHs are chemical compounds comprised of carbon and hydrogen molecules in a cyclic arrangement, that can be generated from the incomplete combustion of coal, wood, oil, and gas, and through volcanic activity. Humans are exposed to PAHs mainly through inhalation, but also through the ingestion of food contaminated by environmental pollutants or during preparation techniques involving high temperatures (e.g., grilled, smoked, toasted, roasted, and fried foods) [117]. Owing to their lipophilicity, PAHs have a low clearance in the liver, where they are metabolized to epoxides, dihydrodiols, quinones, or phenols, thus producing ROS that cause hepatotoxicity [118]. Animal-based studies have reported that exposure to PAHs such as benzo[a]pyrene (BaP) promotes lipid uptake from blood and lipid biosynthesis in the liver, contributing to NAFLD, oxidative stress, hepatic inflammation, and injury [119,120]. By activating the AhR pathway, BaP causes an overexpression of the estrogen-metabolizing enzyme cytochrome P450 1A1 (CYP1A1), which affects the estrogen signaling pathway, leading to the suppression of FAO and TG export and to the increase in peripheral fat mobilization, resulting in hepatic lipid deposition [120]. Such a mechanism of action of BaP suggests that exposure to this chemical has the potential to affect especially women, who may thus lose the protective effect of estrogen signaling on hepatic steatosis [23,24].

### 3.2. Heavy Metals

“Heavy metals” refer to metals and metalloids with relatively high densities (more than 5 g/cm^3^), that can accumulate along the food chain, and may lead to high toxicity in living organisms [121,122,123]. The group of potentially toxic elements comprises cadmium (Cd), lead (Pb), nickel (Ni), chromium (Cr), mercury (Hg), and metalloids, such as arsenic (As), from both natural sources and industrial activities [123]. It is well known that exposure to xenobiotic metals can cause gastrointestinal, respiratory, cardiovascular, reproductive, renal, hemopoietic, and neurological disorders [123]. The oxidative stress caused by these metals, by rising levels of oxidative damage in a cell, destroys lipids, proteins, and DNA molecules, and supports carcinogenesis. Exposure to environmental heavy metals, especially through ingestion and inhalation, plays a role in the development of NAFLD even in lean men [124,125,126,127,128,129,130,131,132,133].

**Figure 3:**
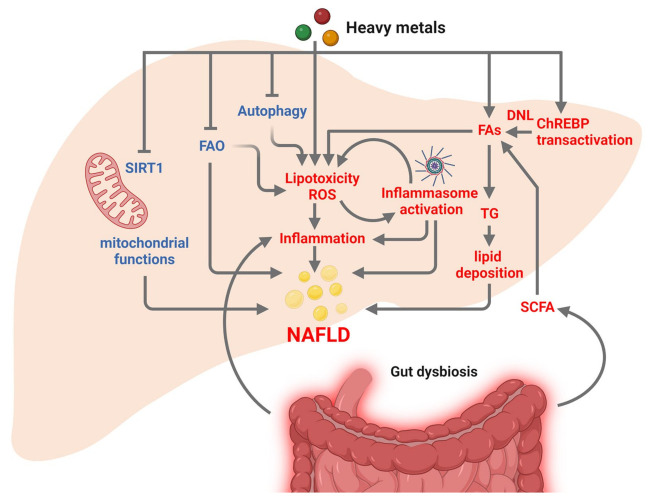
Major effects of heavy metals on the main pathways involved in NAFLD development. Dietary intake of heavy metals leads to impaired regulation of lipid metabolism, by promoting lipid synthesis and deposition and by inhibiting lipid catabolism. Heavy metals enhance DNL also through the increased transactivation of ChREBP, favoring TG synthesis and lipid deposition. Heavy metals impair mitochondrial functions and inhibit SIRT1, FAO, and autophagy, resulting in increased production of ROS and lipotoxicity, which in turn activate NLRP3 inflammasome and boost inflammation, further favoring NAFLD progression. Heavy metals contribute to NAFLD pathogenesis also by affecting gut permeability, thus increasing the flux of SCFA and inflammatory molecules toward the liver. The pathways/factors that increased or decreased are shown in red or blue, respectively. Abbreviations: ChREBP: carbohydrate responsive element binding protein; DNL: de novo lipogenesis; FAs: fatty acids; FAO: fatty acid oxidation; NLRP3: NLR family pyrin domain containing 3; ROS: reactive oxygen species; SCFA: short chain fatty acids; SIRT1: sirtuin 1; TG: triglyceride. Figure created with BioRender (https://biorender.com/, accessed on 11 May 2023).

Industrial emissions, smoking, and consumption of contaminated food and water represent the main sources of Cd, another toxic heavy metal widely disseminated in the environment [121,134]. In the body, Cd accumulates mainly in the liver, where it causes hepatotoxicity [126,134]. Several population-based studies indicate that environmental Cd is a tangible risk factor for NAFLD [125,127,128]. Elevated urinary Cd levels have been found to correlate with markers of NAFLD/NASH, hepatic necroinflammation, and cytokine levels [129,135]. In rodent models, Cd exposure leads to fatty liver and non-specific chronic inflammation, apoptosis, and liver cell regeneration, facilitating NASH development [136,137,138]. In other mouse models of NAFLD, chronic exposure to low doses of Cd alters HFD-associated adverse health effects, by limiting or exacerbating lipid deposition and liver injury dependent on Cd concentration [139]. Once taken up from the bloodstream, Cd interacts with the liver through heavy metal binding proteins and leads to impaired regulation of lipid metabolism, marked hepatic mitochondrial dysfunctions along with significant suppression of sirtuin 1 (SIRT1), inhibition of mitochondrial FAO and autophagy [140] (Figure 3).

The main sources of Pb include natural soil enrichment, paint, industrial legacy, batteries, contemporary mining emissions, contaminated food and water, and differ depending on different regional zones [141]. The accumulation through the food chain is the main way of Pb exposure, followed by direct inhalation and skin contact [122]. In a cohort of 41,723 individuals, exposure to Pb resulted to be an independent risk factor for MAFLD (metabolic dysfunction-associated fatty liver disease) [130], a renewed name for NAFLD [142]. In a population-based study of 4582 subjects, blood Pb levels positively correlated with the suspected NAFLD [143]. Chronic Pb exposure during early childhood is associated with hepatic steatosis and hepatocellular injury in young adulthood [144]. Although the mechanism(s) are not fully uncovered, Pb exposure seems to promote NAFLD development and progression by enhancing the production of ROS and oxidative stress, resulting in changes in lipid peroxidation and reduced antioxidant activity in hepatic cells [131]. An interesting study by Daniel et al. showed that Pb exposure may favor NAFLD by lowering the levels of sorcin, a protein that confiscates ChREBP (carbohydrate-responsive element binding protein) within the cytoplasm, thus enhancing the nuclear shuttling and transactivation of ChREBP, that results in increased hepatic DNL [145] (Figure 3). Hepatic Pb-induced liver toxicity may be boosted by the activation of a pro-inflammatory response due to variations in the intercellular signaling between Kupffer cells and hepatic cells [146], or to variations in gut microbiota [147].

Positive correlations have been found between soil heavy metal mixture containing As and the risk of developing NAFLD [86,124,132] as well as between urinary As levels and fatty liver/NAFLD [133]. Exposure to As impairs the normal metabolic features, increases the risk of NAFLD, and induces liver damage and inflammation in mice fed with HFD [148]. As induces the mitochondrial production of ROS, which upregulates the level of mitophagy and oxidizes mitochondrial DNA [149]. In rodents, As promotes NASH due to increased lipid accumulation, autophagy and NLRP3 inflammasome activation, dysregulated expression of lipid-related genes, and alternative cell death processes such as ferroptosis, a type of programmed cell death dependent on iron and characterized by the accumulation of lipid peroxides [150] (Figure 3). As-induced activation of NLRP3 inflammasome promotes the maturation and secretion of pro-inflammatory cytokines such as IL-1β and IL-18 (interleukin 1β and 18), that, in turn, boost hepatic IR and favor NAFLD development [149]. In rat liver, As induces ferroptosis-mediated NASH by affecting the interaction between Mitofusin 2 (a physical tether between the endoplasmic reticulum and mitochondria) and inositol-requiring enzyme 1 alpha (IRE1α) [151].

The combined exposure to several heavy metals can further increase the risk of developing NAFLD and the associated comorbidities [86,131,132,152], further pointing to cumulative effects derived by multiple exposures.

### 3.3. Microplastics and Nanoplastics

In the so-called era of Plasticene, plastics and their derived micro- and nano-particles represent a concern for human health [153]. Microplastics (MPs) are defined as “synthetic solid particles or polymeric matrices, with regular or irregular shape and with size ranging from 1 µm to 5 mm, of either primary or secondary manufacturing origin, which are insoluble in water” [154], while nanoplastics (NPs) are particles smaller than 1 µm [155]. MPs and NPs can be of primary manufacturing origin when deliberately created for consumer and industrial uses, such as occurs for exfoliants in cleansers, cosmetics, drug delivery particles in medicines, and industrial air blasting [155,156]. Otherwise, MPs and NPs can be of secondary origin when derived from the degradation of macroplastics through enzyme-based biodegradation or non-biodegradation processes, such as thermal degradation, physical degradation, photodegradation, thermo-oxidative degradation, and hydrolysis [155,156].

MPs and NPs can occur in both aquatic and terrestrial environments, and eventually enter the human body through the ingestion of contaminated food and water, the inhalation of airborne plastic particles that originate from synthetic textiles and urban dust, and, in a minimal amount, through weakened skin barrier and skin wounds [155,157,158,159]. Among these three routes, the ingestion of plastic-containing food and drinks represents the main route for MPs and NPs to enter the human body, affecting mainly the digestive and secretory systems [155,160]. MPs fragments have been detected in several types of food, including honey, beer, salt, sugar, fish, shrimps, and bivalves, and have been found in 81% of tap and 93% of bottled water, respectively [155]. It has been estimated that the average human is consuming around 39,000 to 52,000 MPs particles per year; people who drink only bottled water can ingest an extra 90,000 particles, further pointing to the food chain as a major source of microplastic consumption by humans [155]. Notably, MPs have been found in stool samples and cirrhotic liver tissues, confirming the exposure of the human digestive tract to MPs [161,162]. Although analytical tools to detect the presence of NPs in food are not yet available, it is conceivable that NPs can occur in the food chain due to the degradation of MPs [155].

Although their widespread distribution, particles in the seafood and the aquatic environment represent the greatest risk of absolute exposure to MPs and NPs and a concern for human health, especially due to the long-term weathering of polymers and the leaching of polymer chemical additives [153,155]. Several chemical additives, including inert or reinforcing fillers, plasticizers, antioxidants, UV stabilizers, lubricants, heavy metals, dyes, and flame-retardants, are added during production to modify plastic qualities such as color and transparency, to enhance the performance of plastic products, and to improve thermal, electrical, and mechanical resistance [153]. Other chemicals can be absorbed by plastic particles from the surrounding environment. In almost all cases, added and adsorbed additives are not chemically bound to the plastic polymer, and, thus, can leach into the air, water, food, and, potentially, the human body [153].

After intestinal absorption or epidermal infiltration, small MPs and NPs enter the bloodstream and reach the liver, where they can be easily internalized through endocytosis or, in an energy-independent way, through passive diffusion in a dose–response manner [157]. Small MPs (25 nm~90 μm) and NPs can accumulate in the livers of marine fish and mammals, leading to hepatic morphological changes, inflammation (necrosis, infiltration), and accumulation of lipid droplets, thereby affecting the normal function of the liver and resulting in hepatotoxicity [155,157].

**Figure 4:**
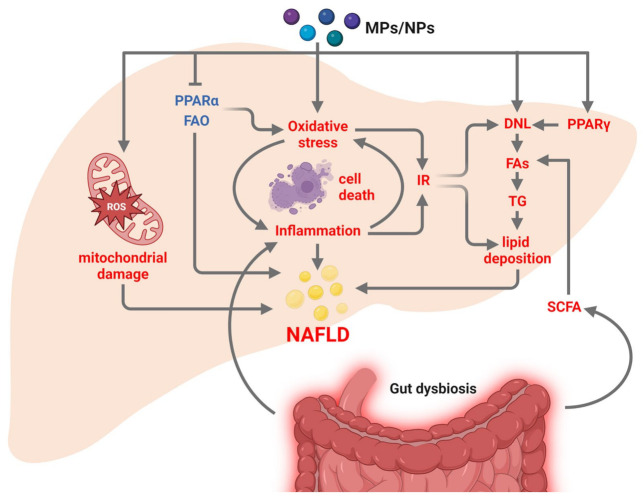
Major effects of MPs and NPs on the main pathways involved in NAFLD development. Dietary intake of MPs and NPs promotes NAFLD by increasing the hepatic expression of PPARγ and their target genes leading to enhanced DNL and TG synthesis, and by reducing FAO and lipid catabolism, mainly through the inhibition of PPARα. Chemical additives associated with MPs/NPs lead to mitochondrial damage, oxidative stress, and inflammation. Oxidative stress and inflammation boost IR, further favoring DNL and lipid deposition. Excessive oxidative stress and inflammation prompt programmed cell death through apoptosis, pyroptosis, and ferroptosis. MPs/NPs-induced gut dysbiosis also contribute to NAFLD pathogenesis by increasing inflammation and the production of SCFA that, once reached the liver, feeds the pool of FAs. The pathways/factors increased or decreased are shown in red or blue, respectively. Abbreviations: DNL: de novo lipogenesis; FAs: fatty acids; FAO: fatty acid oxidation; IR: insulin resistance; MPs: microplastics; NPs: nanoplastics; PPARα: peroxisome proliferator-activated receptor α; PPARγ: peroxisome proliferator-activated receptor γ; ROS: reactive oxygen species; SCFA: short chain fatty acids; TG: triglyceride. Figure created with BioRender (https://biorender.com/, accessed on 11 May 2023).

Increasing evidence suggests that ingested or inhaled MPs and NPs damage liver health [155,157] and facilitate NAFLD development and progression [163,164,165] (Figure 4). Oral exposure to polystyrene MPs induces metabolic disturbances, such as diabetes and NAFLD, especially in mice fed with HFD, mainly due to inflammation of the intestinal mucosa that impairs nutrient absorption [164]. In mice fed with HFD, polystyrene NPs potentiate liver damage and trigger the development of hepatic fibrosis, by interfering with liver lipid metabolism, lowering superoxide dismutase (SOD) activity, inducing oxidative stress, inflammation, and collagen fiber deposition [165]. Excessive production of ROS activates the PI3K/Akt (phosphoinositide-3-kinase/protein kinase B) signaling pathways, blocking insulin signal transduction, and leading to IR, which may account for the increased plasma glucose levels and liver lipid droplets in mice following long-term and low-dose oral administration of polystyrene NPs [166]. In human hepatic cells, the uptake, intracellular localization, and cytotoxic effects of polystyrene NPs depend on particle concentration and surface functionalization and induce profound metabolic changes, especially in mitochondrial-related processes, accounting for mitochondrial damage [167,168,169]. In human liver organoids, 1 μm polystyrene MPs microbeads have been found to disrupt lipid metabolism by increasing the expression of HNF4A, alter ATP production, promote ROS generation, oxidative stress by inducing CYP2E1 (cytochrome P450 family 2 subfamily E member 1), inflammation, lipotoxicity, and hepatotoxicity, thus providing evidence for the implication of these particles in human NAFLD [170].

From a mechanistic point of view, MPs/NPs promote liver lipid deposition by altering the hepatic expression of genes involved in lipid metabolism, such as PPARα and PPARγ and their target genes, leading to enhanced DNL (i.e., higher mRNA levels of *Fasn*, *Srebp1*) and lipid transport (i.e., higher mRNA levels of *Cd36*) and to a reduction of lipid catabolism (i.e., lower mRNA levels of *Cpt1α*, carnitine palmitoyltransferase 1A) and PPARα-mediated lipolysis [157,165,171]. MPs/NPs-associated chemical additives may prompt the excessive production of ROS, leading to lipid peroxidation and oxidative damage [157,163,170,172]. Compared to MPs, NPs easily aggregate in living organisms, favoring a stronger oxidative stress response and liver damage [157]. Exposure to MPs/NPs increases the expression and activities of inflammatory factors such as IL-1β, TNFα, and NF-κB, the infiltration in the liver, and polarization of macrophages toward an M1 phenotype, further aggravating liver injury [157]. Excessive oxidative stress and inflammation prompt ER stress and programmed cell death through apoptosis, pyroptosis, and ferroptosis [157,173,174,175].

Transcriptomic and metabolomic analysis has revealed that several biological processes related to energy metabolism, including glycolysis/gluconeogenesis, glucose transport, pentose phosphate pathway (PPP), fatty acid synthesis, and oxidation, are inhibited in the livers of mice and fish exposed to MPs [157,163,169]. Inhibition of the PPP pathway reduces the levels of the bio-reductant NADPH [176], further boosting oxidative-dependent cell damage. In fish, MPs more than NPs affect the energy metabolism, causing changes in feeding activity, depletion of the energy reserves, disturbances in the ability to mobilize energy reserves, defects in the mitochondrial membrane respiratory chain, and low levels of ATP/ADP/AMP in the liver [157,169,170].

Exposure to MPs and NPs may also enhance the expression and activity of cytochrome P450 oxidases (CYP450s, i.e., CYP1A1, CYP11A1, CYP19A1, CYP2E1) mainly involved in phase I reactions of oxidation, reduction, and hydrolysis, and may increase the activities of alkaline phosphatase (ALP), aspartate aminotransferase (AST), alanine aminotransferase (ALT), that are released into the blood during liver injury [157].

MPs and NPs damage intestinal function favoring gut dysbiosis and gut permeability and impair the balance of gut microbes leading to changes in the production of short-chain fatty acids, fatty acyl chains, choline, cholesterol, and AA, which in turn contribute to NAFLD pathogenesis favoring liver steatosis, inflammation, and fibrosis [163,164,177,178,179].

Although few studies investigated the sex differences associated with MPs/NPs exposure, evidence suggests that liver metabolic and toxic effects may be worse for females [180,181], given the major impact of these plastic particles and their associated chemical additives (i.e., EDCs) on female reproductive parameters and functions [181,182,183,184].

### 3.4. Air Particulate Matter

Particulate matter (PM) refers to solid particles and liquid droplets that are discharged into the air as a result of diesel use, road and agricultural dust, coal and biomass combustion, and emissions from industrial activities [185]. PM is composed of microscopic carbonaceous particles and the adsorbed chemicals, such PAHs, aryl hydrocarbons, volatile organic hydrocarbons, heavy metals (especially Fe, iron; Cu, copper; Ni), organic compounds, minerals, inorganic ions, and biological materials [185]. PM is classified depending on particle diameter: PM_10_ (<10 μm), PM_2.5_ (<2.5 μm), PM_1_ (<1 μm), and PM_0.1_ (<0.1 μm), also termed ultrafine particles (UFPs). The toxicity of specific subclasses of inhaled PM depends on particle size (with the smaller ones being the more toxic) and shape and on the composition of organic/inorganic fractions of PM as well as chemicals adsorbed to particle surface [186,187]. Being more abundant than UFPs, PM_2.5_ is considered to be the most harmful to human health [185].

In the respiratory compartment, <PM_2.5_ particles dissolve in the aqueous lining and reach the alveoli, where they induce the activation of macrophages towards an activated phenotype, triggering local and systemic inflammation [185]. Once crossed the alveolar barrier, <PM_2.5_ might enter the systemic circulation and, in a secondary phase, reach the liver, where it could be internalized via endocytosis-mediated mechanisms and favor metabolic dysfunctions and hepatic inflammation [188,189,190,191]. Three-week exposure to PM_2.5_ leads to the development of low-grade liver inflammation and enhances pro-inflammatory cytokines in plasma, while long-term exposure to “real world” PM_2.5_ leads to several molecular and metabolic derangements, including the rise in plasma TG, low/very low-density lipoproteins *ratio* (LDL/VLDL), IR, altered glucose metabolism, and a more sustained inflammatory response [191,192]. In rodent models, PM_2.5_ exposure synergizes with unbalanced dietary regimens (i.e., HFD) and aggravates metabolic and inflammatory dysfunctions, accelerating NAFLD progression to NASH [188,193].

From a mechanistic point of view, PM_2.5_ exposure can inhibit the expression of PPARα and PPARγ, impairing the regulation of glucose and lipid metabolism, FAO, and immune response, thus leading to hepatic steatosis, inflammation, and IR [194]. PM-induced metabolic effects are, indeed, mediated at least in part by the downregulation of PPARα and their lipid metabolism-related target genes in the liver (i.e., *Cyp4a14*; *Cd36*; *Slc27a1,* solute carrier family 27 member 1) and in BAT (i.e., *Ucp1*, uncoupling protein 1), which favor hepatic steatosis and BAT whitening [194]. In mice, exposure to PM_2.5_ for 16 weeks decreases glycolysis and Krebs cycle intermediates, while increasing the incorporation of 13C in the oxidative branch of the pentose phosphate pathway, suggesting that this metabolic shift can support de novo synthesis of fatty acids prior to the onset of IR [195]. PM_2.5_ treatment induces lipid synthesis in human hepatic HepG2 cells as well [196].

Both short-term acute and long-term chronic exposure to PM_2.5_ stimulate inflammatory cells and induce local tissue and systemic inflammation by increasing the levels of pro-inflammatory cytokines (IL-1β, IL-18, IL-6, and TNF-α) and liver injury associated with dyslipidemia [191]. In the liver, PM-induced cytokines activate Kupffer cells and promote inflammation through the activation of several molecular pathways, including c-JNKs-activator protein 1 (AP1), toll-like receptor 4 (TLR4), and NF-κB, thus favoring NASH development [197]. Additionally in adipocytes, together with impaired glucose tolerance, IR, and mitochondrial alterations, PM_2.5_ may prompt the expression of pro-inflammatory factors, such as TNFα and IL-6, and reduce anti-inflammatory factors, such as IL-10, further contributing to the propagation of systemic inflammation [198]. In turn, pro-inflammatory cytokines such as IL-6 may aggravate hepatic IR by inducing the activation of the STAT3/SOCS3 (signal transducer and activator of transcription 3/suppressor of cytokine signaling 3) pathway [199].

Prolonged PM_2.5_ exposure affects liver health also through the generation of ROS, which elevates the risk of oxidative stress-driven NAFLD by triggering lipid accumulation in the liver [192]. ROS production can also be mediated by heavy metals, especially Cr(VI), Pb, and As, contained within or adsorbed to particles as well as by electrophilic reaction metabolites derived from organic compounds attached to the surface of particles [200]. PM_2.5_ -induced production of ROS may reduce cell antioxidant capacity by impairing the translocation of nuclear transcription factor NRF-2 (nuclear factor erythroid 2–related factor 2) to the nucleus, thus affecting the expression of their target genes and the activity of antioxidant enzymes such as SOD, CAT (catalase), GPX (glutathione peroxidase), further propagating the oxidative stress [192,201]. Excessive ROS production and insufficient antioxidant activity may damage hepatic cells by altering the structure and function of biological macromolecules such as DNA, proteins, and lipids and by activating NF-κB, apoptosis, JNK, and p53 signaling pathways [191]. In turn, PM_2.5_-induced activation of inflammation can boost the production of ROS and reactive nitrogen species [191,192], further fostering oxidative stress. Oxidative stress derived from long-term exposure to PM_2.5_ can cause ER stress, that, in turn, aggravates liver lipid accumulation and IR through the selective activation of the unfolded protein response (UPR) signaling pathways, thus accelerating the progression of NAFLD [202,203].

PM_2.5_ exposure can promote NAFLD development and progression even as a consequence of changes in the intestinal microflora and gut dysbiosis [20,191,204,205]. Altered intestinal microflora may, indeed, increase the production and secretion of harmful substances, especially LPS [204]. Once entered the blood and reached the liver through the portal vein, intestinal bacteria-derived toxic derivatives may intensify a pro-inflammatory response, further exacerbating NAFLD progression [191,206]. Exposure to high doses of PM promotes a pro-oxidative and pro-inflammatory response and tight junction damage, leading to the death of gastrointestinal epithelial cells and to increased intestinal permeability [205]. In diet-induced mouse models of NAFLD, as well as in NAFLD patients, intestinal barrier dysfunctions and altered intestinal permeability may facilitate the transfer of LPS into the systemic circulation, exacerbating liver inflammation and the progression of NAFLD toward NASH and fibrosis [20,207,208].

Evidence suggests that long-term exposure to PM_2.5_ might have different metabolic effects in the two sexes [209]. Compared to their male counterparts, female mice showed greater IR, increased levels of hepatic TG, free fatty acids (FFA), and cholesterol (CH), and enhanced hepatic expression of ApoB (apolipoprotein B) and MTTP [209]. The greater vulnerability of females towards PM_2.5_ can be due to the inhibition of the hypothalamus–pituitary–adrenal (HPA) axis and to the decreased glucocorticoids levels, which may contribute to IR and to the disorders of hepatic lipid metabolism [209].

In a rodent model of menopause, the lack of estrogen action predisposes females to PM-negative effects by altering metabolic, oxidative, pro-inflammatory, and heat shock protein levels [210], further pointing to the greater susceptibility towards air pollution of females with unbalanced hormonal and reproductive systems.

## 4. Climate Change, Food Insecurity, and NAFLD

Although very poorly investigated, even climate change and global warming as consequences of environmental pollution may further account for the increasing incidence of NAFLD worldwide [211]. Global warming, indeed, threatens agriculture production leading to food insecurity and shortage of food supplies and favors the consumption of processed and imported foods with little nutritional value, thus accounting for an increased risk for humans to develop metabolic disorders, including NAFLD [211,212,213,214].

Several studies have shown that global warming favors the growth rate and production of microcystins (MCs), the most common class of liver toxins produced as secondary metabolites by a number of widely distributed freshwater cyanobacteria [215,216,217]. MCs are cyclic heptapeptides with two conventional amino acids in positions X and Y and a unique β-amino acid ADDA (3-Amino-9-methoxy-2,6,8-trimethyl-10-phenyldeca-4,6-dienoic acid) [215,218]. Based on their hydrophobicity and ability to form a chemical bond between the toxin and the protein phosphatases within cells, the two conventional amino acids differently contribute to MC toxicity and cell damage [218]. Among over 300 different MCs identified to date, microcystin-LR (MC-LR, so-called because it contains amino acids leucine (L) and arginine (R) in the X and Y positions, respectively), is the most abundant, persistent, and toxic MC variant, categorized as group 2B carcinogen by the International Agency for Research on Cancer (IARC) [218].

Once synthesized, MCs are stored intracellularly and only released into the water following cell lysis, either by a viral infection or during cell senescence. Human exposure to MCs occurs through the chronic and accidental ingestion of contaminated drinking or recreational water, inhalation or contact with the nasal mucous membrane, dermal contact with toxins during recreational activities, and consumption of contaminated food (i.e., vegetables, fruit, fish, and shellfish) irrigated with or grown in contaminated water [219]. Although the majority of MCs is eliminated with the feces, part of MCs is absorbed in the intestine and distributed to other organs, in particular to the liver [219], where MCs can be easily uptaken due to the higher expression of the MC organic anion transporting polypeptides (OATPs), especially *Oatp1b2* as demonstrated for the mouse liver [218]. Once inside and taken up by the cells, the toxins promote cell damage by specifically inhibiting the serine/threonine protein phosphatases (PP)-PP1 and PP2A, leading to protein hyperphosphorylation, and alterations in the cytoskeleton, and increasing oxidative stress, cell death, cytoskeleton disruption, and cell lysis [219,220].

Being the liver the primary target organ for MC toxicity [218,219], several epidemiological and pre-clinical studies found a positive relationship between MCs and biomarkers of liver damage as well as dysfunctions in glucose, triglyceride, lipid, and cholesterol metabolic pathways [218,219,221,222,223,224,225]. Long-term environmental exposure to MCs increases the risk of NAFLD in humans [221] and exacerbates hepatic injury in rodent models of NAFLD [222,223,224,226], by increasing the expression of genes related to fatty acid biosynthesis and uptake, oxidative stress, pro-inflammation, necrosis, fibrosis, collagen deposition, hepatotoxicity, and by reducing the expression of genes involved in fatty acid β-oxidation, lipoprotein transport, and anti-inflammatory response. The relationship between MC exposure and NAFLD seems to be bi-directional since it has been reported that NAFLD may alter MC-LR toxicokinetics and acute toxicity [227]. Interestingly, MC-LR’s impact on liver functions in mice is different between the two sexes, with females showing higher susceptibility to MC-LR compared to males [228].

The increase in mean temperature consequent to global warming may promote NAFLD development even through the reduction of BAT activity with an impact on energy expenditure and thermogenesis [229,230]. Limited capacity and functionality of BAT might furthermore alter the regulation of glucose and lipid metabolism [231,232], triggering metabolic dysfunctions (i.e., increased adiposity, IR, T2D, and gestational diabetes) [229,233,234,235] and NAFLD development [236,237] due to the cross-talk between the BAT and the liver [231,238,239]. Environmental-induced BAT dysfunctionality may differently account for NAFLD susceptibility in the two sexes, as suggested by increasing evidence on sexual dimorphic and hormone-dependent regulation of BAT metabolism [240,241,242,243,244].

## 5. Dietary Intake of Environmental Pollutants, Female Subfertility, and NAFLD

**Figure 5:**
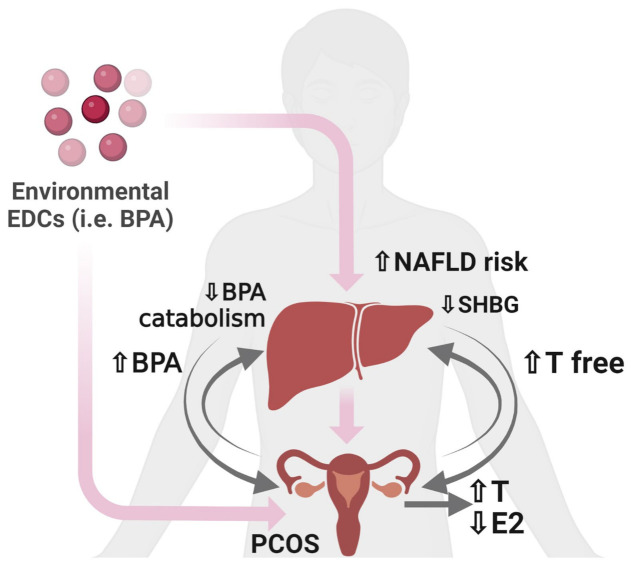
Exposure to environmental endocrine pollutants such as BPA increases the susceptibility of PCOS women to develop NAFLD. In ovarian theca cells, BPA enhances the production of androgens, further impairing T/E2 *ratio*. At the hepatic level, BPA decreases SHBG, thus contributing to high levels of free T. In the liver, androgens downregulate BPA liver catabolism, thereby increasing circulating BPA levels. Abbreviations: BPA: Bisphenol A; E2: estradiol/estrogens; PCOS: polycystic ovary syndrome; SHBG: sex hormone-binding globulin; T: testosterone/androgens. Figure created with BioRender (https://biorender.com/, accessed on 11 May 2023).

Given the strict relationship between reproduction and the regulation of energy metabolism in female mammals [245], exposure to environmental pollutants may be detrimental, especially in women with reproductive dysfunctions; conversely, acting as EDCs, environmental pollutants may promote reproductive dysfunctions, further favoring metabolic disorders. A canonical example is represented by PCOS, an endocrine and metabolic condition affecting 5–18% of women, which diagnosis—according to the 2003 Rotterdam criteria—is confirmed with two of the three following criteria: clinical or biochemical hyperandrogenism, irregular cycles, and polycystic ovary morphology [246,247]. PCOS women show an increased susceptibly to the development of metabolic dysfunctions, including fatty liver, NAFLD, and NASH [248,249,250], likely a consequence of altered hormone levels (increased androgens and androgens/estrogens *ratio*; decreased estrogens) and IR [251,252,253]. Beyond genetic and epigenetic susceptibility, hypothalamic and ovarian dysfunctions, exposure to high androgen levels, IR, increased adiposity, and obesity [246,247], environmental pollutants have a role in PCOS physiopathology [254,255,256,257,258,259] and, thus, may further contribute to NAFLD incidence in PCOS women (Figure 5).

Exposure to BPA, the main component of plastic containers, enhances the production of androgens in ovarian theca cells and affects their hepatic metabolism by interacting with SHBG (sex hormone-binding globulin) and with enzymes regulating their hydroxylation at the hepatic level [254]. Androgens *per se* can downregulate BPA liver catabolism, increasing its circulating levels and, thereby, promoting PCOS [254].

A positive association has been found between PCOS and heavy metals, especially Cr, Cu, As, Cd, Pb, and Hg [258,260]. Acting as EDCs, heavy metals impair hypothalamic–pituitary–gonadal (HPG) axis, promote androgen synthesis, and several inflammatory and metabolic alterations, including the generation of oxidative stress [258,260]. In PCOS women, indeed, serum As, Cd, Pb, and Hg levels are increased and negatively correlated with serum levels of glutathione (GSH) and SOD [260].

Women exposed to high concentrations of fine air pollutants, including PM_2.5_, had an increased risk of developing PCOS [259]. Organic solvents found in indoor decoration may activate TNFα as well, and lead to excessive hepatic glucose production, low muscular glucose uptake, and impaired insulin sensitivity, further increasing metabolic and ovulatory dysfunctions in women suffering from PCOS [261].

## 6. Maternal Exposure to Pollutants and Developmental Origins of NAFLD

**Figure 6:**
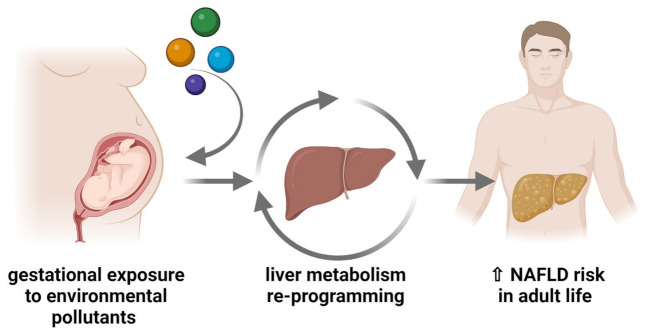
Gestational exposure to environmental pollutants acting as EDCs interferes with liver metabolism programming and increases the risk of developing NAFLD later in life. Figure created with BioRender (https://biorender.com/, accessed on 11 May 2023).

The epidemic and still growing prevalence of NAFLD worldwide [1] cannot be merely explained by changes in diet, lifestyle, and environmental factors that occurred in the last few decades, nor from their association with genetic risk factors that should have undergone few changes in such a limited time frame. In addition to these risk factors, it is conceivable that transgenerational effects may account for the developmental origins of NAFLD [25], contributing to the spreading of this pathology. There is a growing body of evidence showing that prenatal exposure to endocrine disruptors may alter liver metabolic programs and represent a risk factor for NAFLD development later in life [262,263] (Figure 6). In this view, prenatal exposure to environmental pollutants acting as endocrine and/or metabolic disruptors may contribute to or aggravate NAFLD development and progression, as suggested by several studies in rodents [264,265,266,267,268,269,270,271,272] and some clinical studies [273,274,275].

Gestational exposure to BPA induces fatty liver development in male offspring rodents by altering the expression and activity of several nuclear transcription factors [264,265,266]. Prenatal exposure to BPA promotes lipid deposition in the liver of offspring male mice through impairment of NR signaling, such as the inhibition of *Hnf1b* (hepatocyte nuclear factor 1b) and the upregulation of *Pparγ* [264]. In obese and diet-induced rodent models of NAFLD, perinatal exposure to BPA leads to the induction of NRF2 signaling, aggravates NAFLD onset, and exacerbates NAFLD progression toward a NASH-like phenotype [266,267,268]. The consumption of drinking water containing BPA during gestation impairs the PI3K/Akt/mTOR (PI3K/Akt/mammalian target of rapamycin) and TLR4/NF-κB pathways in the liver of offspring rats, resulting in the upregulation of lipogenic genes, activation of the inflammatory response, dysregulation of autophagy and development of NAFLD [265]. Possibly because the majority of pre-clinical studies have been conducted in males, it has not been clarified whether prenatal exposure to BPA might lead to sex differences in the offspring. In fact, while some studies have shown similar effects in the liver of both sexes [265], other studies have reported that gestational and developmental exposure to BPA leads to sexually dimorphic changes in hepatic gene expression and epigenome at birth and may exacerbate HFD-induced hepatic steatosis in a sex-specific fashion [269,270]. Perinatal exposure to endocrine-disrupting pollutants with estrogenic activity may, indeed, have a greater impact on male offspring, likely interfering with the liver metabolic programming prompted by estrogen signaling through organizational effects at this developmental stage [28].

In humans, higher exposure to PFAAs during pregnancy has been found to be associated with increased susceptibility to liver injury in children, who show higher serum levels of ALT, AST, and GGT (gamma-glutamyltransferase), and increased serum levels of aromatic AA and, especially, BCAA [273], the last ones having a role in NAFLD onset and progression [276,277,278,279].

Beyond direct effects on the liver, fetal exposure to environmental pollutants such as triphenyl phosphate (an organophosphate flame retardant) and nitenpyram (an insecticide used in agriculture) can trigger NAFLD and NASH development later in life as a consequence of impaired intestinal dysbiosis and colonic mucosal damage [271,280].

In utero exposure to heavy metals, possibly acting as EDCs, such as As and Hg increases offspring susceptibly to NAFLD, IR, liver inflammation, and injury [272,274].

In mice, gestational exposure to polystyrene NPs induces hepatic steatosis in the dams and in adult female offspring but not male offspring, by enhancing the expression of genes involved in DNL, FA uptake, and TG synthesis [281].

Maternal exposure to ≤PM_2.5_ predisposes adult male mice offspring to NAFLD and other long-term metabolic dysfunctions such as obesity, T2D, insulin resistance, hypertension, hyperlipidemia, and metabolic syndrome, likely affecting organogenesis and tissue functions [282,283,284,285]. In mice, prenatal exposure to diesel exhaust PM_2.5_ (DEP) leads to increased lipogenesis and worsens fatty acid oxidation, favoring hepatic lipid deposition in the adult male offspring [286]. In humans, the association between prenatal exposure to ≤PM_2.5_ and the risk of developing NAFLD later in life is more consistent, likely due to the very few population-based investigations completed [282]. Nevertheless, accordingly, with the multiple-hits hypothesis of NAFLD pathogenesis [287], some studies support the idea that early life PM_2.5_ exposure induces liver damage, especially in overweight/obese children [275]. Interestingly, prenatal DEP exposure has been shown to relieve hepatic steatosis and liver function in the offspring of mice fed with HFD, suggesting a complex interaction between nutritional and environmental conditions in prompting NAFLD [286]. Although very little investigated, maternal exposure to PM_2.5_ seems to have sex-specific effects in the offspring with a more severe impact on females, which show increased hepatic expression of markers of oxidative stress and inflammation [288].

## 7. Discussion

The prevalence of NAFLD and its associated diseases has become a significant health and economic burden nowadays and it is still rising. Although the well-known role of EDCs in NAFLD development, the specific contribution of dietary intake of environmental endocrine pollutants to the increasing incidence of NAFLD still represents a significant gap in our knowledge. Although rodent studies have provided the strongest evidence for this cause–effect association, only a small percentage of these studies have been focused on females, limiting the understanding of the molecular mechanisms through which females can be eventually more vulnerable to dietary exposure to environmental pollutants depending on their hormonal status. Given the strict interplay between metabolism and reproduction in females, dietary intake of contaminated food and water can be, indeed, particularly detrimental to the liver health of females with impaired hormonal signaling and reproductive dysfunctions. Conversely, environmental EDCs can trigger reproductive alterations in females, that, in turn, facilitate NAFLD development and progression. In addition to that, dietary intake of environmental endocrine pollutants during gestation can be a risk factor for developing NAFLD for the mothers as well as for the offspring, likely affecting the estrogen-driven programming of liver metabolism especially in males. Given this evidence, additional rodent studies are warranted to expand our knowledge of the consequences of dietary intake of environmental endocrine pollutants, with the aim to elucidate the underlying mechanisms of action and to identify valuable biomarkers and interventional strategies for the treatment of NAFLD.

## Data Availability

All data are publicly available.
